# Knowledge Mapping of Aspergillus-Related Research in Respiratory Medicine: A Bibliometric Analysis Based on English-Language Articles Spanning 1975 to 2022

**DOI:** 10.1155/carj/6199860

**Published:** 2024-11-27

**Authors:** Fan Jiang, Xuemei Li, Qing Qiao, Ge Zhang, Yanmei Zhang, Lin Su

**Affiliations:** ^1^Respiratory Medicine Department, The Fourth People's Hospital of Jinan, Jinan 250031, Shandong, China; ^2^Section of Health, No. 94804 Unit of the Chinese People's Liberation Army, Shanghai 200434, China; ^3^Resident Standardization Training Cadet Corps, Air Force Medical Center, Beijing 100142, China

**Keywords:** Aspergillus, bibliometrics, bibliometrix, Citespace, VOSviewer

## Abstract

**Background:** Aspergillus has become the second most common causative agent of invasive fungal infections and is the leading cause of death from fungal infections.

**Methods:** English-language publications ranging from 1975 to 2022 collected from the Web of Science Core Collection database were analyzed visually using VOSviewer, R package Bibliometrix, Scimago graphic, Gephi, Pajek, and Microsoft Excel 365. Literature search using the advanced search function in WoSCC with the search formula “TS=(Aspergillus).” The inclusion criteria were as follows: (1) article type = article; (2) time span = 1975–2022; (3) topic = “Medical Mycology”; (4) research field = “Respiratory System, Internal Medicine”; (5) language type = English.

**Results:** As of February 21, 2023, studies of Aspergillus in respiratory medicine included 1065 articles with 41,308 cumulative citations. The United States ranked first in terms of the highest number of publications (*n* = 320). Udice French Research University was the institution with the highest number of publications (*n* = 40). The author with the highest number of publications was Denning (*n* = 32). Based on the CiteSpace analysis result, the keywords with the nature of a Burst Citation were “epidemiology,” “chronic pulmonary aspergillosis,” “galactomannan,” “guideline,” “sensitization,” and “randomized trail.” Besides the keywords, it was found that as of 2022, only the topics “allergic bronchopulmonary aspergillosis” and “invasive pulmonary aspergillosis” are still active with a large number of papers published, according to the results of the CiteSpace analysis of the cited literature.

**Conclusions:** The bibliometric results indicated the current hot research hotspots of Aspergillus were as follows: (1) research directly related to Aspergillus (epidemiology, risk factors, early diagnosis methods, and methods of novel antifungal treatment, e.g., galactomannan); (2) studying coinfections of Aspergillus with other pathogens: For example, COVID-19 could focus on studying the immune status of patients with COVID-19-associated pulmonary aspergillosis (CAPA) to guide individualized immunotherapy and individualized management of patients with CAPA.

## 1. Background

Fungi are among the most influential eukaryotic microorganisms on Earth. In recent years, it has been found that fungi are not only prevalent in the natural environment but that many species of fungi also exist in different parts of the human body (including the skin, lungs, genitourinary tract, oral cavity, and gastrointestinal tract), which are part of the normal flora of the human body and play an essential role in human health [1]. However, when the immune system is compromised, commensal fungi can transform into invasive pathogens, leading to invasive fungal infections [2]. Aspergillus is a fungus that significantly affects humans. It has become the second most pathogenic fungus responsible for invasive infections and the leading cause of death from fungal infections [3, 4]. In the past, morbidity rates were low because of inadequate diagnostic methods. In recent years, the morbidity and mortality rates of Aspergillus infection have increased significantly, owing to advances in diagnosis and the addition of new risk factors. New risk factors include an increase in the number of patients undergoing hematopoietic stem cell and organ transplantation; the increasing use of new invasive techniques, broad-spectrum antibiotics, and antineoplastic drugs; and the widespread use of glucocorticoids and immunosuppressive drugs. These factors have led to an increasing trend in the morbidity and mortality associated with invasive Aspergillus infections [5–7]. In addition, the widespread use of antifungal drugs in clinical practice and the misuse of fungicidal drugs in industrial and agricultural production have led to an increase in the number of drug-resistant bacteria, which has also contributed to an increase in mortality. The mortality rate of patients with invasive aspergillosis caused by voriconazole-resistant *Aspergillus fumigatus* in the United States is reportedly 60% [8]. Aspergillus infection is a disease that seriously affects the human quality of life, threatens health, and has become the focus of attention for scientists in the field.

Since Bennett first reported human aspergillosis in 1842, respiratory disease scholars worldwide have carried out extensive work on various aspects of Aspergillus infection, which has deepened our understanding of Aspergillus. However, the growing number of Aspergillus-related research papers is not matched by the lack of a systematic compilation of all existing Aspergillus-related studies. To address this problem and overcome the limitations of systematic reviews in terms of literature quantity, we have attempted to explain the developmental lineage of Aspergillus-related research using bibliometrics, which ultimately serves to predict the frontiers and hotspots of Aspergillus.

Bibliometric visualization software can extract and process citation data to form a visual network map that reduces the workload and facilitates interpretation and analysis. Therefore, in this study, we chose a bibliometric approach to evaluate Aspergillus-related research from an econometric perspective. As proposed by Pritchard in 1969, bibliometrics applies mathematical and statistical methods to study books and other documentary materials. It can systematically analyze the overall development of a research field, identify the research hotspots and trends in the area, and provide a reference for scientific research [9]. Bibliometric visualization software can present collaborative relationships among authors, countries, and institutions, co-occurrence network relationships of research themes and fields, and literature co-citation and coupling analyses to reveal research hotspots, trends, and frontiers. Hence, the data visualization software VOSviewer and CiteSpace are widely used by researchers in different fields and countries as essential tools for bibliometric analysis. This study, on the other hand, is of great significance as the first article in the history of Aspergillus research to apply bibliometrics to categorize and predict the historical lineage of Aspergillus research and future research prospects.

## 2. Methods

### 2.1. Data Resource

Data for this study were obtained from the WoSCC database launched by the Institute for Scientific Information (ISI). Its advantages are as follows: (1) It can provide the literature schema required by the bibliometric analysis software CiteSpace and VOSviewer; (2) compared with PubMed, Scopus, Embase, and other databases, the SCI-E database is the most authoritative and highest-standard database in the world and is widely used by researchers [10]. It includes literature abstracts and other relevant data such as citations and research collaboration information, which are useful for bibliometric analysis. Reference information is provided after acquiring the literature through bibliometric network construction and visualization in VOSviewer and CiteSpace [11].

### 2.2. Retrieval Strategies

**Figure 1:**
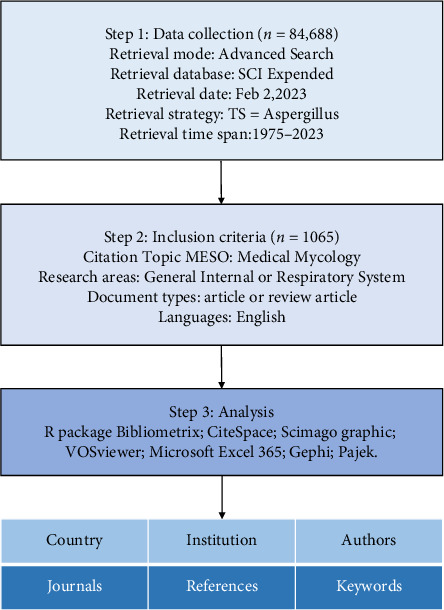
Flowchart of the literature search and selection process.

To ensure the accuracy of the search results, we performed a literature search using the advanced search function in WoSCC with the search formula “TS=(Aspergillus).” The inclusion criteria were as follows: (1) Only “article” was included as the article type; (2) the time span was from 1975 to 2022; (3) the topic included only “Medical Mycology”; (4) only “Respiratory System, Internal Medicine” was selected as the research field; and (5) the language type of the writing must be English [12]. The full information of the search results including title, author's name, affiliation, abstract, keywords, citations, and references was downloaded as txt format files to be further bibliometric analysis [13]. The literature screening process is shown in Figure 1.

### 2.3. Data Extraction

The data were imported into Microsoft Excel 365 (Microsoft Corporation, Redmond, WA, United States) for further analysis. Two researchers (FJ and XML) independently performed the data extraction and literature selection analyses to ensure the reliability of the results. Literature selection refers to the bibliometric analysis, since analysis software needs to set some necessary parameters and thresholds when running, to take into account the rationality of the content and the visualization effect, we need to carry out the continuous adjustment of the parameters to balance the two, and thus, FJ and XML were used the same data for data analysis, respectively [14]. Disagreements were resolved through discussion or third-party negotiation (LS). This study focused on annual publication and citation counts, countries, journals, institutions, authors, citations, and keywords. The WoSCC citation report was used to evaluate H-index and citation frequency. The H-index was used to evaluate academic achievement. The H-index of a researcher/country indicates that the researcher has published h articles, and each article has been cited h times. Thus, it is used to assess the scientific impact and productivity of a researcher or country [15].

### 2.4. Data Visualization and Analysis

Three bibliometric tools, including two software packages (CiteSpace (6.1. R4) and VOSviewer (1.6.18)), and the R package Bibliometrix were used for a more comprehensive analysis. VOSviewer (https://www.vosviewer.com) was developed by Prof. Eck of Leiden University and Prof. Waltman using Java software. VOSviewer evaluates and visualizes study characteristics from different perspectives, such as country/region, keywords, co-authors, research institutions, and co-cited literature [16]. The core idea of the software design is “co-occurrence clustering.” CiteSpace (https://citespace.podia.com) is a computer program developed by Prof. Chen, based on the Java language, that can obtain quantitative information and discover relevant developments and trends in specific scientific fields through load bursts and cluster analysis patterns. Bibliometrix performs a complete set of bibliometric analyses and visualization of results for full bibliometric analysis and visual presentation, statistical analysis, data preprocessing, co-occurrence matrix construction, co-citation analysis, etc. (https://www.bibliometrix.org).

## 3. Results

### 3.1. Search Results and the Status of Aspergillus Research

**Figure 2:**
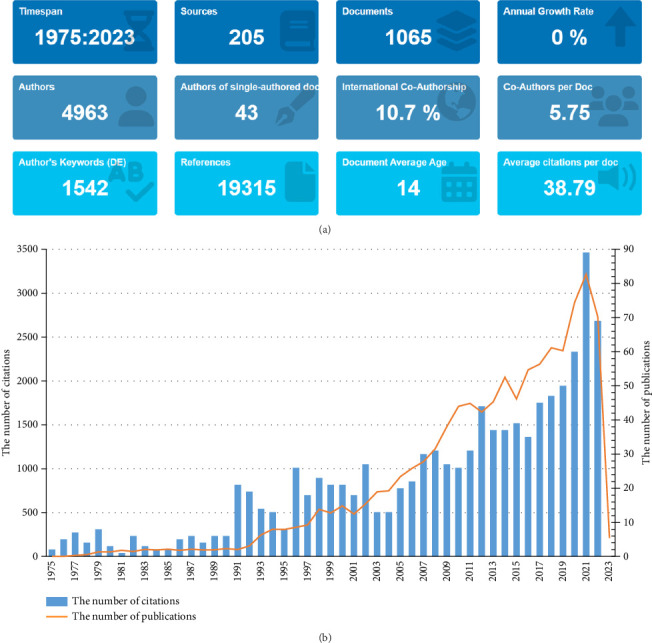
The global trend of annual publications and citations related to Aspergillus in the respiratory system from 1975–2023. (a) Overview of the 1065 included articles on Aspergillus in the respiratory system. Basic information included the time period of included articles, the number of journal categories, the total number of articles, the annual growth rate, the total number of authors, the number of articles published by a single author, the proportion of international co-authors, the number of co-authors of an article, the keywords given by the author, the number of references cited, the average life span of each article, and the cumulative citation times per article. (b) Overall publication trends and citations. The figure shows the overall number of posts and citations in the Aspergillus field from 1975 to 2023.

Using the search strategy shown in Figure 1, we retrieved 1065 original research articles. The current status of Aspergillus research in respiratory medicine using Bibliometrix is shown in Figure 2(a). We analyzed all articles between 1975 and 2023; a total of 205 journals were included. The search results included 1065 articles with 4963 authors, 43 articles written by one author, 10.7% authors with international collaborations, an average of 5–6 authors per article, 1542 keywords provided, and 19,315 references cited. The average lifespan of each paper, that is from being noticed to becoming unknown, was 14 years, and each article was cited an average of 23–24 times.

### 3.2. Overall Trend in the Number of Publications and Citations

Analysis of the variation in the number of publications with respect to the year provides insights into the development rate of the Aspergillus research field. As reported in the citation analysis based on the WOS database (Figure 2(b)), there are two phases in the curve: a steady growth phase from 1975–1993 and a rapid growth phase from 1992–2021. There was a significant increase in the frequency of citations in the literature from 2020–2021, indicating that research on Aspergillus in the respiratory system is still a concern as well as a field with good research prospects. According to the analysis of the WOS database, the total number of articles published in all research literature related to Aspergillus in respiratory medicine was 1065, and the total citation frequency was 41,308, with an average of 38.79 citations per article and an H-index of 90. In addition, we display the details of the top 10 countries, institutions, journals, and authors in Table 1.

### 3.3. Analysis of Published Articles by Country

**Figure 3:**
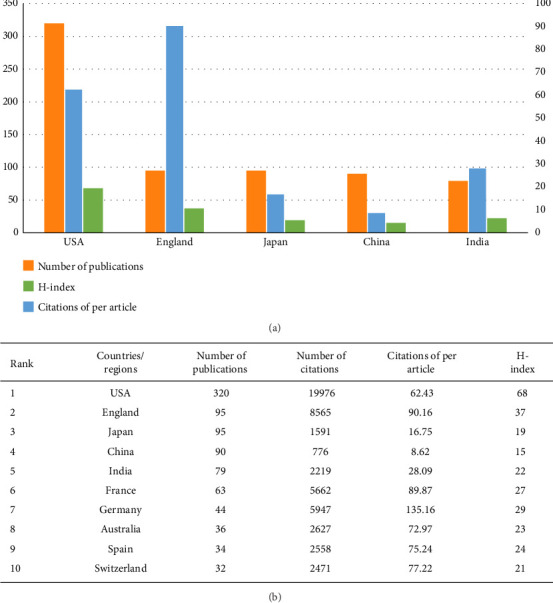
Current status of national research output. (a) Countries in the top five international rankings for research on Aspergillus in the respiratory system. Countries ranked in the top five in the world are evaluated according to the number of publications, the H-index, and the cumulative number of citations per article. (b) The top 10 countries/regions contributing to research on Aspergillus in the respiratory system. Countries ranked in the top five in the world are evaluated according to the number of publications, number of citations, the H-index, and the cumulative number of citations per article.

Collaborations between countries and institutions play an important role in facilitating the exchange and dissemination of knowledge among scholars. Consequently, we conducted a co-occurrence network analysis of the countries where papers were published using the VOSviewer software to understand the collaboration between countries in Aspergillus research in respiratory medicine. First, we summarized the publications of the top 10 countries/regions. The United States ranked first in terms of research productivity (number of publications: 320; percentage of publications: 30.047%), indicating the outstanding scientific value of the United States in Aspergillus in clinical research. The United States was followed by the United Kingdom (UK) (95, 8.92%), Japan (95, 8.92%), China (90, 8.451%), and India (79, 7.418%). After excluding self-citations, the United States had 19,976 citations and an H-index of 68, placing it in the top position, followed by the UK, Japan, China, and India, with total citations and an H-index of 8565, 2665, 1591, and 776, and 37, 19, 15, and 22, respectively (Figures 3(a) and 3(b)).

**Figure 4:**
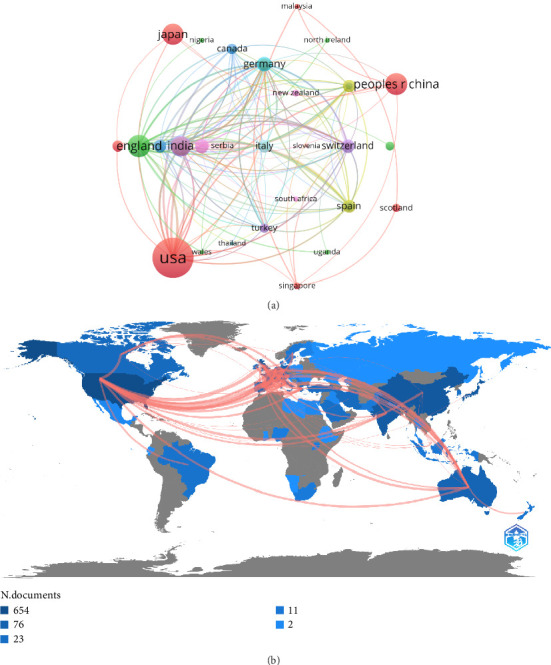
Current status of international cooperation. (a) Visual maps of international cooperation between countries involved in Aspergillus research. (b) Geographic distribution map displaying the global distribution of research in Aspergillus in the respiratory system. Different countries/regions are denoted in different colors based on the number of articles published.

Only countries with a minimum number of publications were included in the network. Only the 50 countries that met the threshold were analyzed using the VOSviewer (Figure 4(a)). The selected 50 countries were divided into 15 clusters according to the degree of cooperation, with the three larger clusters being as follows: Cluster 1 (red) mainly includes the United States, China, Japan, Iran, Scotland, and Singapore; Cluster 2 (green) primarily includes England, Saudi Arabia, Nigeria, and Northern Ireland; Cluster 3 (blue) includes Belgium, Canada, Israel, South Korea, and Thailand. The United States, England, China, Japan, and Iran have many publications, which are indicated in the figure with a larger circle area. The geographical distribution map based on the total number of publications from each country is shown in Figure 4(b).

### 3.4. Analysis of the Institutions

**Figure 5:**
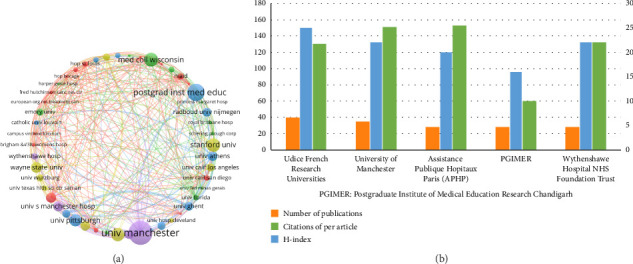
Output and collaboration status of Aspergillus research institutions. (a) Visual map of the current status of interagency cooperation regarding research on Aspergillus in the respiratory system. Different colors represent different cooperative groups; the connection indicates collaboration and the connection width indicates the degree of cooperation. (b) Top five global institutions in Aspergillus research in the respiratory system. The ranking is based on the number of publications, the H-index, and the cumulative number of citations per article.

To further understand the research status and actual contributions of issuing institutions to the field of Aspergillus in the respiratory system, the number of articles issued by each research institution was counted. A total of 1065 articles were screened and imported into VOSviewer, and the parameter was set to a minimum number of citations for an institution of 622 articles. Out of the 1478 institutions, 50 met the inclusion criteria. Based on the degree of cooperation, the 50 institutions were divided into six clusters, as shown in Figure 5(a): (1) red cluster: University of Texas at Austin, National Institute of Allergy and Infection Diseases, University of California San Diego, Hautepierre Hospital, Hôpital Saint-Louis; (2) green cluster: Medical College of Wisconsin, Emory University, the Medical University of Vienna, University of Florida; (3) blue cluster: Postgraduate Institute of Medical Education and Research (PGIMER), University of Pittsburgh, University of Athens, Radboud University, Nijmegen, and Ghent University; and (4) yellow cluster: Stanford University, the University of Minnesota, Wayne State University, and Paris Descartes University. In addition, we present the top five institutions according to the number of publications in Figure 5(b). During the specified study period, 1446 institutions published articles on Aspergillus in respiratory medicine. The institution with the highest number of publications was Udice (40 publications; 3.756%), the largest contributor to the field. This was followed by the University of Manchester, UK (35; 3.286%), and Assistance Publique Hopitaux Paris (APHP) (28; 3.2629%). These core research institutions were universities or well-known hospitals, indicating that most research on Aspergillus is conducted in hospitals and universities. In terms of the average number of citations per article, Udice French Research Universities (202) > University of Manchester (*n* = 161) > APHP (*n* = 159) > PGIMER (*n* = 152) > Wythenshawe Hospital NHS Foundation Trust (*n* = 150). Regarding the H-index: Udice French Research Universities (25) > University of Manchester (22) = APHP (22) > PGIMER (20) = Wythenshawe Hospital NHS Foundation Trust (20).

### 3.5. Analysis of Journals and Co-Cited Journals

**Figure 6:**
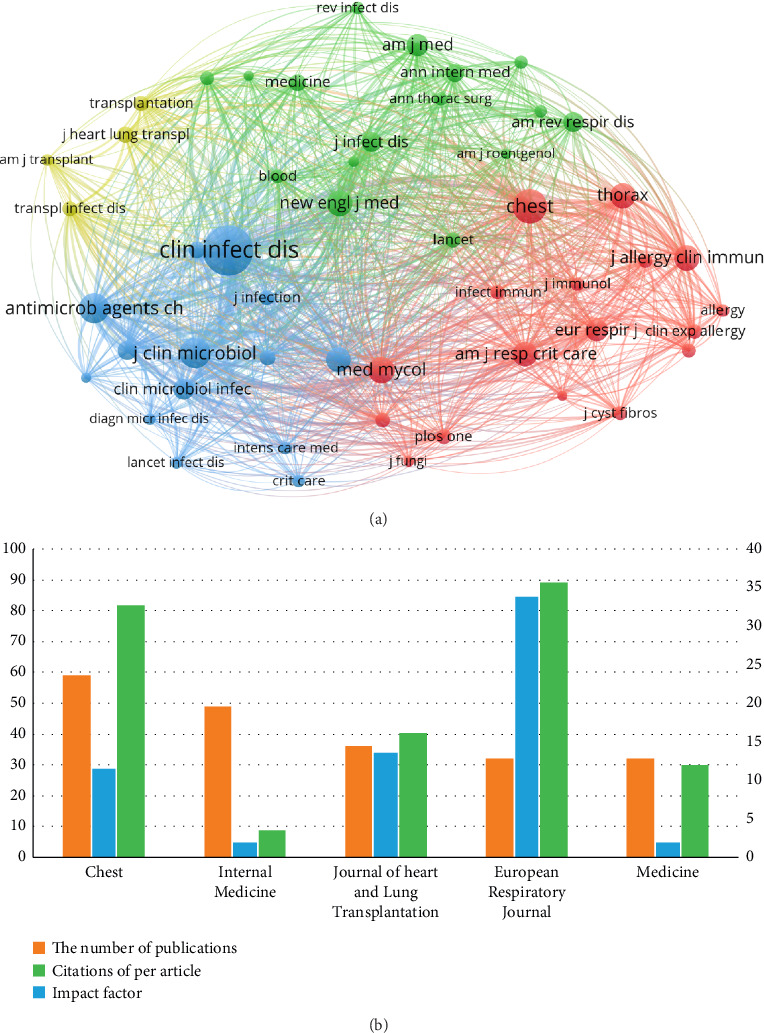
Journal output and keyword co-occurrence in the Aspergillus research field. (a) Network visualization of journals based on VOSviewer. This network map groups keywords with close relationships into single clusters, each denoted by a distinct color. All the keywords could be divided into five clusters: Cluster 1 (red nodes), Cluster 2 (blue nodes), Cluster 3 (green nodes), and Cluster 4 (yellow nodes). (b) The five journals with the most significant number of publications. The top five international publications are evaluated regarding the number of publications, the H-index, and the impact factor.

As a vehicle for disseminating scientific knowledge, journals promote discipline development and academic communication. An analysis of co-citation between journals from 1975–2023 was performed by VOSviewer, and the co-citation network of journals consists of four clusters corresponding to the four colors (Figure 6(a)). The top three journals in terms of number of citations were Clinical Infectious Diseases (3027), Chest (1402), and Journal of Clinical Microbiology (1155). All 51 kinds of journals are excellent JCR1 region journals, and they were divided into four clusters. The four larger clusters were as follows: Cluster 1 (red) mainly included Chest, Medical Mycology Journal, Thorax, Journal of Allergy and Clinical Immunology, and American Journal of Respiratory and Critical Care Medicine; Cluster 2 (blue) primarily included Clinical Infectious Diseases, Journal of Clinical Microbiology, Antimicrobial Agents and Chemotherapy, and Mycoses; Cluster 3 (green) included the New England Journal of Medicine, American Journal of Medical Genetics, American Review of Respiratory Disease, The Journal of Infectious Diseases, and Annals of Internal Medicine; and Cluster 4 (yellow) mainly included the Journal of Heart and Lung Transplantation, Transplantation, and Transplant Infectious Disease.

This study encompassed a review of articles published in 206 journals relevant to the field. Of the 1065 articles analyzed, 311—accounting for 29.2% of the total—were published in the top 10 most active journals in the field of Aspergillus research related to respiratory issues. The top five journals are listed in Figure 6(b). The highest number of relevant articles was published by Chest (59), followed by Internal Medicine (49), and the Journal of Heart and Lung Transplantation (36). In terms of citations per article, Chest ranked first (*n* = 89), followed by Internal Medicine (*n* = 81), the Journal of Heart and Lung Transplantation (*n* = 40), European Respiratory Journal (*n* = 29), and Medicine (*n* = 8). Regarding H-index, the order was as follows: Chest (33.801) > Internal Medicine (13.569) > Journal of Heart and Lung Transplantation (11.393) > European Respiratory Journal (1.82) > Medicine (1.817).

### 3.6. Analysis of Authors and Co-Cited Authors

**Figure 7:**
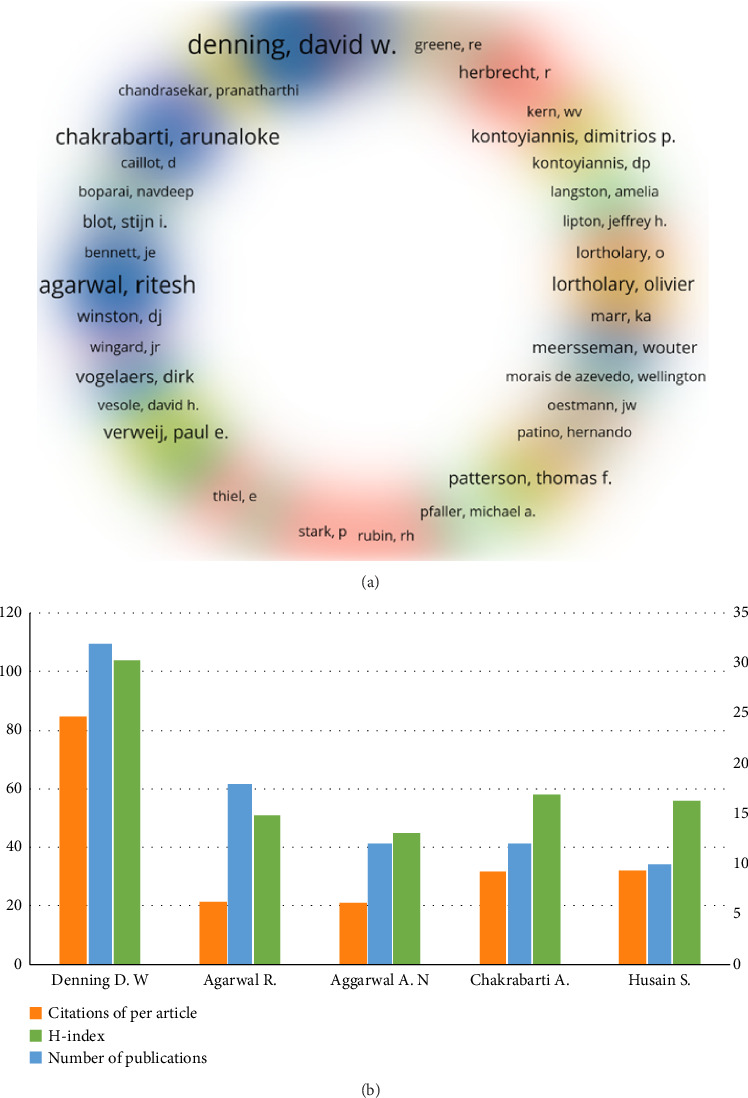
Output and cluster analysis of researchers. (a) Co-citation and cluster analysis of researchers. The size of the researcher's name is closely related to the number of publications, with the same color indicating the same number of co-citations. (b) The top five international researchers are evaluated on the number of publications, the H-index, and the cumulative number of citations per article.

The number of research papers published by an author translates into a contribution to research in the field. Four thousand nine hundred and eighty-eight researchers authored the 1065 articles included in this study. Utilizing VOSviewer to assess author collaborations from 1975 to 2023 and setting the minimum citation count for inclusion at 624, we found that only 50 of the 5262 authors met this benchmark (Figure 7(a)). The selected 50 authors were divided into 7 clusters according to the degree of collaboration. Among the three largest clusters, Cluster 1 (red) included Denning D.W., Agarwal R, Chandrasekar P.H., Herbrecht R, and de Pauw B. Denning, D.W., and Agarwal R had many publications in Aspergillus respiratory research. Cluster 2 (yellow) mainly included Kontoyiannis, DP, Dr. Amelia Ann Langston, MD, and Jeffrey H Lipton from the United States. Cluster 3 (green) primarily included Stijn Blot, Chakrabarti Arunaloke, and Dimopoulos G.  Figure 7(b) shows the top five most prolific authors focusing on Aspergillus in respiratory research over the past 47 years. The first was Denning D.W. [*n* = 32] from the University of Manchester, UK, who published the most papers, followed by Agarwal R (*n* = 18), Aggarwal AN (*n* = 12), Chakrabarti A (*n* = 12), and Husain S (*n* = 10), H-index: Denning D.W. (104) > Chakrabarti A (58) > Husain S (56) > Agarwal R (51) > Aggarwal A.N. (45). Our findings showed the absolute dominance and influence of the author Denning D.W. in respiratory Aspergillus.

### 3.7. Analysis of Keywords

#### 3.7.1. Analysis of Co-Occurrence

**Figure 8:**
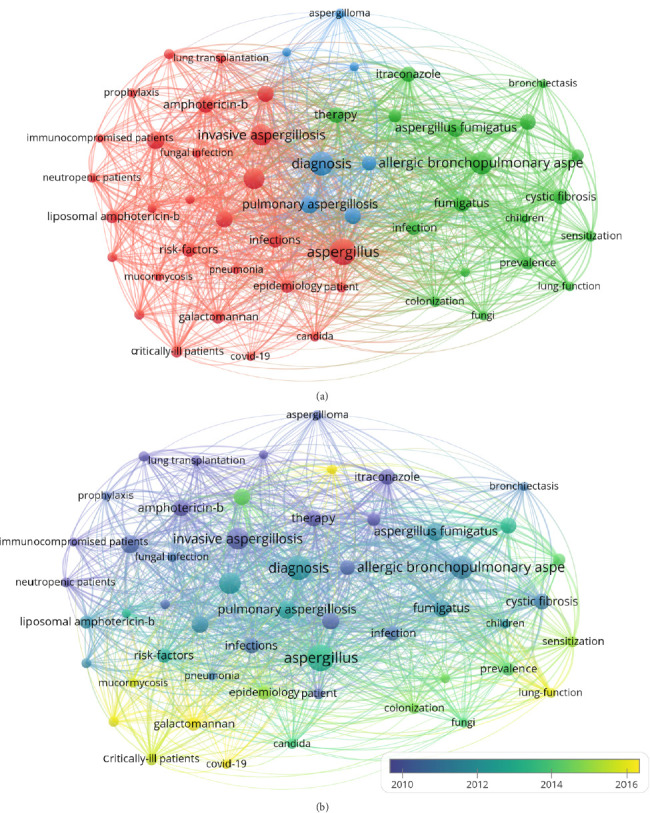
Analysis of keyword co-occurrence. (a) Network visualization of keywords based on VOSviewer. In this network map, keywords with close relationships are assigned to one cluster with the same color. All the keywords could be divided into five clusters: Cluster 1 (red nodes), Cluster 2 (green nodes), Cluster 3 (blue nodes). (b) Overlay visualization of keywords based on VOSviewer. The nodes marked with purple or blue represent the keywords that appeared relatively earlier, whereas those coded with yellow represent current research focuses.

Keywords are distilled from the research content of the article, and through statistical and co-occurrence analyses of the keyword frequency of the article, current research hotspots in the field can be effectively derived. Understanding the hotspots of big data research, the frontiers of development, and conducting corresponding research will have important guiding significance. Network visualization was generated by applying VOSviewer to keywords that co-occurred more than 20 times between 1975 and 2023 (Figure 8). The five keywords with the highest number of co-occurrences were “aspergillus,” “diagnosis,” “allergic bronchopulmonary aspergillosis,” “invasive aspergillosis,” and “aspergillosis.” Cluster 1 is represented in green and contains 28 keywords: “amphotericin-b,” “antifungal therapy,” “aspergillosis,” “aspergillus,” “bronchoalveolar lavage,” “bronchoalveolar lavage fluid,” “candida,” “covid-19,” “critically-ill patients,” “epidemiology,” “fungal infection,” “fungal-infections,” “galactomannan,” “galactomannan,” “infections,” “invasive aspergillosis,” “invasive fungal-infections,” “invasive pulmonary aspergillosis,” “liposomal amphotericin-b,” “lung transplantation,” “mucormycosis,” “neutropenic patients,” “patient,” “pneumonia,” “prophylaxis,” “recipients voriconazole,” “risk-factors,” “voriconazole.” Cluster 2, represented in red, contains the keywords: “allergic bronchopulmonary aspergillosis,” “aspergillus fumigatus,” “aspergillus-fumigatus,” “asthma,” “bronchiectasis,” “children,” “colonization,” “cystic-fibrosis,” “fumigatus,” “fungi,” “infection,” “itraconazole,” “lung-function,” “prevalence,” “sensitization,” “therapy.” Cluster 3, represented in blue, contains the keywords: “aspergilloma,” “chronic pulmonary aspergillosis,” “diagnosis,” “disease,” “management,” “pulmonary aspergillosis,” and “spectrum.”

#### 3.7.2. Burst Citation Analysis

**Figure 9:**
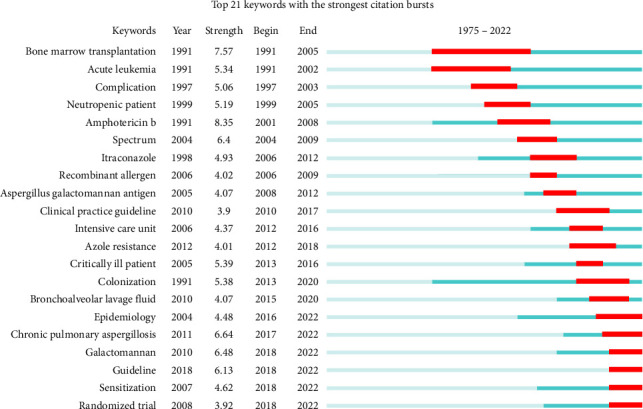
Burst keywords. Top 25 keywords experiencing significant bursts in citation frequency in the Aspergillus research field.

We utilized CiteSpace to analyze the keywords exhibiting bursts in publications about Aspergillus in the context of respiratory medicine (Figure 9). We list the top 21 keywords with the highest number of citations. The blue line represents the period from 1975 to 2022, and the red line represents the cycle of each burst keyword. The most cited keywords before 2000 were as follows: “bone marrow transplantation,” “acute leukemia,” “complication,” “neutropenic patient,” and “amphotericin b.” The keywords that had citation bursts after 2022 were as follows: “epidemiology” (2016–2022), “chronic pulmonary aspergillosis” (2017–2022), “galactomannan” (2018–2022), “guideline” (2018–2022), “sensitization” (2018–2022), and “randomized trial” (2018–2022).

### 3.8. Co-Citation and Clustering Analysis of References

**Figure 10:**
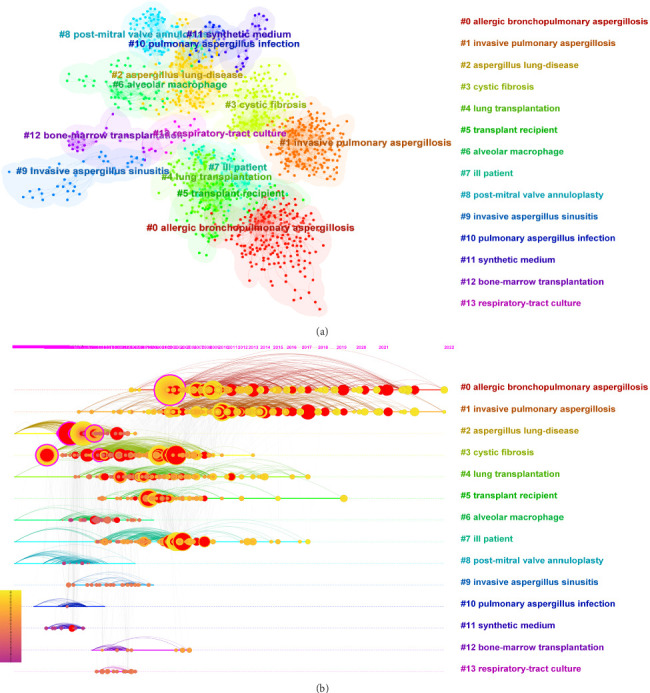
Content distribution and time variation of references. (a) The co-citation of references reflects which articles were studied in the same direction. Cluster analysis classifies the references related to Aspergillus in the respiratory system research in the past 50 years into 14 clusters using the maximum likelihood ratio (LLR) method. Combined with calculating the degree of centrality and the transfer of references with high centrality on the time axis, we can analyze how the research process of the discipline changes and what representative references are available at each stage. (b) Clustering analysis on the timeline view. Circles indicated references; the size of the diameter of the circle was closely related to the number of citations; purple circles reflect the existence of significant turning points in scientific knowledge, i.e., higher centrality; the purple axis indicated 1975–2022.

Co-citation analysis can help classify the literature based on clustering characteristics and understand the main components of the current research field (Figure 10). The co-citation of the literature reflects articles studied in the same direction. Cluster analysis was performed using the largest likelihood ratio (LLR) method to classify the literature related to Aspergillus in the field of respiratory research over the last 47 years, which can be roughly divided into 14 clusters. The 14 clusters included the following: #0 allergic bronchopulmonary aspergillosis (ABPA), # 1 invasive pulmonary aspergillosis (IPA), #2 aspergillus lung-disease, #3 cystic fibrosis, #4 lung transplantation, #5 transplant recipient, #6 alveolar macrophage, #7 ill patient, #8 postmitral valve annuloplasty, # 9 invasive Aspergillus sinusitis, #10 pulmonary aspergillus infection, #11 synthetic medium, #12 bone marrow transplantation, and #13 respiratory tract culture. After understanding the composition of the research content, we analyzed the distribution of different research content over time. We performed a clustering and centrality analysis of the literature through CiteSpace to identify the main research content for each period from 1975–2022 (Figure 10(b)). Finally, considering that the 10 most cited articles are also of interest to most researchers in the field of Aspergillus in respiratory research, we have listed them in Table 2.

## 4. Discussion

To our knowledge, this is the first bibliometric approach to scrutinize the literature related to Aspergillus in respiratory medicine. A total of 165 papers and their associated 19,315 citations from 206 journals, 1446 institutions, and 66 countries met the criteria for analysis. Using bibliometric analysis techniques and visualization tools, we examined countries and regions, research institutions, journals, authors, references, keywords, and other relevant content. We summarized the historical lineage of research on Aspergillus-related studies in respiratory medicine and predicted cutting-edge hotspots in the field.

Based on Figure 2, the total number of articles related to Aspergillus in respiratory medicine in the WoSSC SCI-E from 1975 to 2023 was 1065. Over the past 47 years, the number of publications has gradually increased, suggesting that Aspergillus species are highly valued in respiratory medicine. The curve was divided into two stages. Most articles were clinical studies conducted during the stable growth stage from 1975 to 1993, including the discovery of Aspergillus infection in patients undergoing bone marrow transplantation, acquired immune deficiency syndrome (AIDS), neutropenia, postoperative heart surgery, and primary lung cancer. This highlights the importance of Aspergillus infection in immunocompromised patients. However, a limited understanding of fungi and the absence of new antifungal drugs at the time resulted in a relatively small number of publications. The second stage, beginning in 1992, showed a significant increase in publications, owing to the discovery of antifungal drugs such as fluconazole and itraconazole in the late 1980s.

Additionally, new diagnostic methods have emerged, such as the use of the Aspergillus antigen to detect and diagnose invasive aspergillosis, which has also contributed to an increase in publications [17]. In 2002, there was a significant increase in publications and citations, which can be attributed to a major event in the field. In 2001, a multicenter study compared the efficacy, safety, and tolerability of oral voriconazole and fluconazole in 391 immunocompromised patients diagnosed with esophageal candidiasis, using a randomized, double-blind, double-simulation design. This trial demonstrated that voriconazole was as effective as fluconazole in treating biopsy-confirmed esophageal candidiasis in immunocompromised patients [18]. In 2002, a multicenter, open-label, noncomparative study evaluated the efficacy and safety of voriconazole in patients with invasive aspergillosis and demonstrated its effectiveness in treating acute IPA [19]. These results led to the approval of voriconazole by the US Food and Drug Administration (FDA) for marketing in May 2002. This approval has stimulated the interest and enthusiasm of pharmaceutical personnel and clinical researchers. Notably, there was a significant increase in publications and citations in 2021 due to the outbreak of COVID-19. As COVID-19 damages the human respiratory system and the treatment process often involves the use of steroids and immunosuppressants, many researchers anticipated a risk of fungal infection in COVID-19 patients [20, 21]. As a result, there has been a significant increase in related articles domestically and internationally [22–24].

Figure 3 illustrates the correlation between a country's research output and its strength in respiratory medicine and Aspergillus research. The United States has significant research strength in these fields, owing to its long-standing biomedical foundation and substantial financial support. According to online data, the National Institutes of Health will receive a research budget of 34.8 billion US dollars in 2022. Financial support plays a significant role in the promotion of scientific research. The University of Texas at Austin, the US Department of Veterans Affairs, and the Veterans Health Administration (VHA) are among the top 10 research institutions in the world and are all located in the United States.  Figure 4 shows that the United States cooperates closely with other countries in scientific research, which is beneficial. Moreover, the United States is a global leader in pharmaceutical research and development. For example, Pfizer, headquartered in New York, developed two crucial antifungal drugs, fluconazole and voriconazole, while Merck, a US pharmaceutical company, developed the new echinocandin antifungal drug caspofungin. Johnson & Johnson, headquartered in the United States, developed the initial itraconazole. These essential antifungal drugs have facilitated the rapid development of aspergillus research in the United States.

We also noted that two developing countries, China and India, are among the top five countries with the most publications. Developing countries are home to five-sixths of the world's population, primarily distributed in tropical and subtropical regions where fungi thrive. Furthermore, the underdeveloped economies and lack of medical resources in these countries have created difficulties in diagnosing and treating pulmonary aspergillosis [25]. For the aforementioned reasons, developing countries such as China and India are striving to accelerate their medical and health development. The rapid growth of the Chinese economy and increasing demand for medical and health services have resulted in a gradual increase in the country's investment and funding in medicine and health. Nonetheless, China has a lower citation count and H-index. This may be attributed to a late start and weak foundation in biopharmaceuticals, despite substantial investments in medicine and health. Although the number of papers published by China in international journals has considerably increased, the publication of high-quality research papers in top journals is uncommon [26]. In response, Chinese policymakers have encouraged researchers to prioritize the quality of research over its quantity [27].  Figure 4 shows that China has collaborated with many countries, including the United States, Japan, and Singapore. This provides domestic scholars more opportunities to exchange and collaborate with high-level foreign institutions and scholars, helping domestic Aspergillus research remain abreast of the international research frontier in respiratory systems. However, it should be noted that China's cooperation with other countries is not extensive enough. Consequently, China should encourage its institutions to conduct research, strengthen intercountry collaborations, promote the development of relevant fields, and publish high-quality articles.

The top 10 institutions with the highest number of published articles are Udice French Research University, APHP, and University Paris Cite in France, followed by Wythenshawe Hospital NHS Foundation Trust and Hospital in the UK. The list also includes The University of Texas System, the US Department of Veterans Affairs, and the VHA in the United States, followed by Wythenshawe Hospital NHS Foundation Trust in India. These institutions are at the forefront of Aspergillus research in the field of respiratory science. Although France had the highest number of institutions with published articles, it ranked low in terms of the overall number of published articles. France must improve its collaboration with other countries by encouraging cooperation among medical institutions to publish additional articles.

The implementation of institutional research results lies in the publication of scholarly journals.  Figure 6 indicates that Chest is the most productive journal for research about Aspergillus in the respiratory system, boasting the highest impact factor of 33.801 among its contemporaries in the top five. Trailing Chest is Internal Medicine (IF = 13.569) and the Journal of Heart and Lung Transplantation (IF = 11.393). These three journals are excellent JCR1 zone journals that are influential and authoritative in publishing Aspergillus research, increasing their likelihood of being noticed by scholars globally. In the past decade, articles published in these journals have involved the following: (1) investigating invasive aspergillus infection risk factors and fungal infection prevention, and early diagnosis methods for high-risk hosts, including patients who have undergone heart or lung transplantation, COVID-19 infection, and malignant tumors; (2) exploring the efficacy and safety of nebulized antifungal therapy; (3) research on cystic fibrosis in patients with Aspergillus infection. Furthermore, some articles have mentioned trends and predictions related to influenza-associated Aspergillus, which can serve as a guide for Aspergillus research.

Journal articles serve as platforms for researchers to demonstrate their accomplishments. In this study, we analyzed the top researchers in the field of Aspergillus research in the respiratory system. Denning, from the University of Manchester in the UK, had the highest number of publications and H-index in this field. His research team has the most significant research strength and influence, making it likely to publish significant findings related to Aspergillus research in the respiratory system. By examining the research areas of various authors, researchers can quickly identify potential collaborators and produce high-quality articles. Our clustering analysis (Figure 7) reflects this finding. For instance, the primary collaborative group in Cluster 1 consists of Denning D.W. and Agarwal R, both of whom have a high number of publications in Aspergillus respiratory research (Figure 7), which may be due to their close collaboration. In Cluster 2, Kontoyiannis D.P., a researcher from the United States whose primary focus is on the treatment of leukemia, myelodysplastic syndromes, lymphoma, graft-versus-host disease (GVHD), aplastic anemia, and HIV-related patients, is present alongside Jeffrey H Lipton, whose expertise lies in allogeneic stem cell/bone marrow transplantation, bone marrow failure, and chronic myeloid leukemia (CML). Since both authors study diseases related to the hematopoietic system and have a similar patient population, their research articles on Aspergillus were grouped. Strengthening cooperation in the same research field will promote mutual progress.

Table 1 displays the top 10 cited references, which directly demonstrate the contributions of outstanding researchers through their publications. A highly cited article indicates significance in a particular field. Among the top 10 cited articles, two were reviews, two were randomized trials, one was a clinical study, and the remaining five were expert consensuses and guideline updates. A clinical study by Rosenberg, published in the Annals of Internal Medicine in 1977, proposed clinical and immunological diagnostic criteria for ABPA and discussed the pathogenesis of the disease [28]. This played a crucial role in the early identification and diagnosis of ABPA, guiding further exploration in the field. Two randomized trials between 2000 and 2005 were identified. The first was a double-blind, placebo-controlled trial of itraconazole for treating ABPA published by D.A. Stevens in the New England Journal of Medicine in 2000 [29]. The author of this article believes that reducing the fungal burden in the respiratory tract will reduce chronic antigen stimulation, decrease inflammatory reactions, and improve symptoms. The trial concluded that the addition of itraconazole in patients with corticosteroid-dependent ABPA could improve the disease without increasing toxicity. This trial is a milestone in the treatment of allergic pulmonary aspergillosis. This indicates that research on ABPA is becoming increasingly refined and personalized. Another trial published by Herbrecht R. and Denning D.W. in the *New England Journal of Medicine* compared voriconazole and amphotericin B for the preliminary treatment of invasive aspergillosis [30]. The study was conducted against the background of amphotericin B being a broad-spectrum and effective antifungal drug for a long time. Still, its use was often limited by safety and poor tolerance. In the 1980s, fluconazole and itraconazole were discovered. While fluconazole was ineffective against Aspergillus, itraconazole was effective against Aspergillus but showed cross-resistance with fluconazole-resistant Candida. These factors have prompted an urgent need to identify highly efficient and safe drugs. Voriconazole and amphotericin B were compared in this trial, and the results showed that voriconazole was superior to amphotericin B in terms of response, survival, and safety. This played a significant role in the successful launch of voriconazole later on. The remaining five updates comprised guidelines or expert consensus, mainly for two types of diseases: (1) revision of the definition [31], diagnosis [32], treatment [33], and management of IPA; (2) definition, pathogenesis, diagnosis [34], and management of ABPA. This indicates that research on Aspergillus-related diseases constantly deepens, becoming more refined and individualized.

Since the onset of the novel coronavirus in 2019, there has been a notable surge in literature concerning COVID-19 patients concurrently afflicted with pulmonary aspergillosis. Through investigating these studies, we can conclude the hot spot of pulmonary aspergillosis and COVID-19 as follows: (1) further investigation into the risk factors, precise definition, adaptable diagnostic criteria, and optimal management strategies for COVID-19-associated pulmonary aspergillosis (CAPA); (2) delving into the immune profiles of continuous positive airway pressure (CPAP) patients post-COVID-19 infection, intending to inform personalized immune therapies and appraise the repercussions of immune modulation on patient outcomes; (3) determining the most opportune timing for commencing antifungal therapy, assessing its efficacy, and establishing the appropriate treatment duration for CAPA patients; and (4) identification and effective management of post-COVID-19 sequelae in infected patients. In general, this is the first in-depth study of patients with viral–fungal coinfections, highlighting the growing significance of pulmonary aspergillosis in human health.

The central idea of an article can be conveyed through keywords, and analyzing their distribution over time can provide an overview of the research hotspots during that period. In Figure 8(b), keyword clustering revealed three main categories. The first cluster, represented in red, focuses on invasive aspergillosis, including host conditions, risk factors, diagnostic methods, epidemiology, and treatment. The green cluster focuses on ABPA, encompassing susceptible populations, sensitization, impact on lung function, and treatment. The blue cluster concentrates on chronic aspergillosis, pulmonary aspergilloma, and the diagnostic criteria. Figure 8 shows the temporal distribution of the cluster analysis results, revealing keywords that emerged after 2015, such as COVID-19, chronic pulmonary aspergillosis (CPA), beta-glucan, bronchoalveolar lavage fluid (BALF), lung function, mucormycosis, and critically ill patients. Notably, the diagnosis of critically ill patients and the role of beta-glucan in the diagnosis seem to be recent research hotspots in IPA. The impact of ABPA on lung function and treatment has become a research hotspot, with COVID-19 being the most recent keyword. These findings are consistent with those in the literature and indicate a new direction in the field of Aspergillus respiratory medicine.

The burst keyword analysis results (Figure 9) revealed that the burst keywords in 2000 include “bone marrow transplantation,” “acute leukemia,” “complication,” “neutropenic patient,” and “amphotericin B.” During this period, the research focused on the discovery stage of Aspergillus, explicitly recognizing the fungus's susceptibility to infect patients with low immune function. At that time, antifungal drugs were limited to amphotericin B. In contrast, the emergent keywords in 2022 were “epidemiology” (2016–2022), “chronic pulmonary aspergillosis” (2017–2022), “galactomannan” (2018–2022), “guideline” (2018–2022), “sensitization” (2018–2022), and “randomized trial” (2018–2022). This indicates that the research on Aspergillus infections has expanded to include epidemiology, pathogenesis, diagnosis, and treatment.

An initial report on IPA identified it as a progressive and often fatal disease that primarily affects patients with hematological malignancies and is usually only detectable during autopsies [35]. Over the following decades, IPA has emerged as a severe infectious disease in various populations, including patients with neutropenia, bone marrow or stem cell transplant recipients, solid organ transplant recipients, and patients with chronic granulomatous disease [36]. In 2008, the consensus definition for invasive fungal diseases was updated to include host factors such as solid organ transplantation, congenital immune deficiencies, connective tissue diseases, and immunosuppressive therapy [31]. Later, studies found that ICU admission or severe influenza were risk factors for developing IPA [37–40]. As research on aspergillosis progressed, other diseases were identified as risk factors for IPA, such as chronic obstructive pulmonary disease (COPD) [41], long-term use of steroids [42], chronic liver disease [43], chronic kidney disease [44], drowning [45], and diabetes [46]. In 2018, a multicenter cohort study found that patients with influenza often had comorbid IPA, making influenza an independent high-risk factor for IPA [37].

Furthermore, steroids are increasingly recognized as essential risk factors for invasive fungal infections [47]. The COVID-19 outbreak in 2019 has attracted the attention of researchers. Patients with underlying diseases that require intensive treatment, including glucocorticoids, immunosuppressive agents, mechanical ventilation, and other measures, are at an increased risk of developing fungal infections due to the destruction of their immune function [48]. Therefore, impaired immune function is not the only host factor of aspergillosis. Many studies have shown that aspergillosis can occur in immunocompetent patients without the typical risk factors. Conducting epidemiological investigations of pulmonary aspergillosis and better defining high-risk patient populations is crucial.

The emergence of antifungal drug resistance is a challenge in the treatment of pulmonary aspergillosis [49]. In some European regions, the azole resistance rate approaches 30%, whereas in areas outside Europe, it ranges from 0.6% to 11.2% [50]. Therefore, patients with pulmonary aspergillosis may require long-term drug therapy. However, the use of existing antifungal drugs is limited by various factors, such as the spectrum of activity, drug concentrations in tissues and organs, toxicity, drug interactions, and resistance. The emergence of multi–drug-resistant strains has made the situation even more severe [49]. Immunotherapy is a promising approach for the treatment of fungal infections, including aspergillosis. Immunotherapeutic methods include cell-mediated immunotherapy, such as the infusion of innate cells (neutrophils, dendritic cells, and natural killer cells) and adoptive T-cell transfer; the use of various cytokines and chemokines, such as G-CSF, GM-CSF, M-CSF, IFN-*γ*, and TNF-*α*, and vaccination with fungal antigens [51]. In a 2005 study by Sonov et al., the use of caspofungin or liposomal amphotericin B combined with G-CSF to treat aspergillosis in mice increased the survival rate to 78.9%. It reduces the fungal burden on the organs [52]. Bandera et al. reported three cases of IPA treated with a combination of IFN-*γ* and GM-CSF, which showed increased peripheral blood leukocyte counts and Th1 responses and achieved good efficacy [53].

Although the preliminary exploration of immunotherapy for aspergillosis has achieved good results, its limitations lie in animal experiments and clinical studies, which require further research to validate its feasibility. New antifungal drugs currently being tested in clinical trials include drugs targeting fungal cell wall integrity, cell membrane metabolic pathways, novel CYP inhibitors, and drugs targeting cell signaling pathways. For example, olorofim selectively inhibits the key enzyme dihydroorotate dehydrogenase (DHODH) involved in fungal pyrimidine synthesis, rapidly inhibits the germination and hyphal elongation of Aspergillus at low concentrations, and causes cell swelling and rupture after long-term exposure, showing strong antibacterial effects against cryptic and azole-resistant Aspergillus strains [54]. Rezafungin acts by inhibiting 1,3-*β*-D-glucan synthase, targeting the integrity of the fungal cell wall. A multicenter, double-blind, double-dummy, randomized Phase 3 trial compared the efficacy and safety of rezafungin and caspofungin in the treatment of candidemia and invasive candidiasis (ReSTORE), and showed the effectiveness and safety of rezafungin [55]. Hérivaux et al. found that the diversity of the airway microbiome could predict the onset of IPA. They emphasized its potential as a diagnostic and therapeutic target for fungal respiratory diseases [56]. Therefore, future research on aspergillosis should focus on antifungal therapies, including mechanisms underlying antifungal drug resistance, new fungal treatment methods, and other related fields.

Delay in early diagnosis remains a significant hurdle in the successful treatment of Aspergillus infections. Beta-glucan (GM) is a specific biomarker of Aspergillus infection in clinical settings [57]. The enzyme-linked immunosorbent assay (ELISA) is the most sensitive method for detecting GM antigens. GM antigen detection in BALF is valuable for the early diagnosis of IPA [58]. Diagnosing Aspergillus IgG antibodies in serum is the most reliable method for diagnosing CPA [59]. However, antifungal prevention and treatment can impair the detection performance of routine blood screening, thereby reducing its specificity and sensitivity. In the LFIA-based fungal antigen detection method, the Aspergillus-specific lateral flow device (LFD) uses the JF5 monoclonal antibody to detect the cell surface glycoprotein (mannan) antigen secreted by active Aspergillus species. A recent meta-analysis of a retrospective clinical study showed that LFD performed better on BAL samples than on serum [60]. Moreover, imaging techniques have advanced early diagnosis of Aspergillus infections. Immuno-PET/MRI, which combines the analytical capabilities of positron emission tomography (PET) and magnetic resonance imaging (MRI) with illus-specific radiolabeled antibodies, is expected to improve the specificity and diagnostic performance of IPA diagnosis [61]. Respiratory testing is an attractive option for diagnosing lung diseases.

In vitro studies of Aspergillus cultures have identified 2-pentylfuran (2-PF) as a volatile metabolite of Aspergillus infection [5, 62]. Two case reports have shown that 2-PF is detectable in the breath of severely immunocompromised patients with IPA and disappears after effective treatment [63]. Antifungal susceptibility testing (AFST) is a promising tool for Aspergillus epidemiological research that helps to monitor the minimum inhibitory concentration (MIC) values of antifungal drug resistance [64]. A newly validated CRISPR/Cas13a technology for the detection of tobacco Aspergillus provides a simple, fast, and affordable test for the diagnosis of tobacco Aspergillus infections [65]. Detection of Aspergillus cfDNA in peripheral blood using metagenomic next-generation sequencing (mNGS) is a rapid and noninvasive diagnostic method for IPA. Combined diagnostic tests significantly improve the detection rate, but no serological diagnosis can reliably diagnose 100% of IA cases. Early diagnostic methods have always been a popular research topic.

By combining high-frequency keywords with emerging keywords, we can infer several future research directions for Aspergillus in the field of respiratory system research: (1) epidemiology and risk factors of pulmonary aspergillosis infection, especially IPA; (2) treatment of Aspergillus infection; and (3) early diagnostic methods.

The limitations of this study are as follows: (i) The data analyzed in this article are only from WOS and do not include literature from other databases, such as Scopus and PubMed; (ii) recently published articles (2023) were not included because of delays in WOS database updates; (iii) VOSviewers and CiteSpace do not provide advanced statistical analysis functions; (iv) because the software and R package used for bibliometric analysis only support English, we have referred to the following four articles which were also analyzed by screening English articles only; and (v) nonassessment of the methodological quality of the articles analyzed. Nevertheless, this article effectively describes the global trends in Aspergillus research in the field of respiratory system research. This study also has the following advantages: (i) the first bibliometric research paper on Aspergillus-related studies in the respiratory system, which is a reference for subsequent researchers to conduct their studies and find potential collaborators; (ii) it provides a new perspective to understand a research area different from systematic reviews and general reviews; and (iii) it is a brave attempt to explore the interdisciplinary exploration of medicine and computer science.

## 5. Conclusions

The bibliometric results indicated the current hot research hotspots of Aspergillus were as follows: (1) research directly related to Aspergillus (epidemiology, risk factors, early diagnosis methods, and methods of novel antifungal treatment, e.g., galactomannan); (2) studying coinfections of Aspergillus with other pathogens, e.g., COVID-19, could focus on studying the immune status of patients with CAPA to guide individualized immunotherapy and individualized management of patients with CAPA.

## Figures and Tables

**Table 1:** Distribution of top countries, institutions, authors, and journals.

**Rank**	**Countries/regions**	**Number of publications**	**Number of citations**	**Citations per article**	**H-index**	**Percentage (*N* = 1065)**

1	USA	320	19,976	62.43	68	30.047
2	England	95	8565	90.16	37	8.92
3	Japan	95	1591	16.75	19	8.92
4	China	90	776	8.62	15	8.451
5	India	79	2219	28.09	22	7.418
6	France	63	5662	89.87	27	5.915
7	Germany	44	5947	135.16	29	4.131
8	Australia	36	2627	72.97	23	3.38
9	Spain	34	2558	75.24	24	3.192
10	Switzerland	32	2471	77.22	21	3.005

**Rank**	**Affiliations**	**Number of publications**	**Number of citations**	**Citations per article**	**H-index**	

1	Udice French Research Universities	40	5213	130.33	25	
2	University of Manchester	35	5278	150.8	22	
3	Assistance Publique Hopitaux Paris (APHP)	28	4276	152.71	20	
4	Postgraduate Institute of Medical Education Research (PGIMER) Chandigarh	28	1684	60.14	16	
5	Wythenshawe Hospital NHS Foundation Trust	28	3694	131.93	22	
6	University Paris Cite	27	4356	161.33	19	
7	University of Texas System	23	4656	202.43	20	
8	US Department of Veterans Affairs	22	1526	69.36	17	
9	Veterans Health Administration (VHA)	22	1526	69.36	17	
10	Wythenshawe Hospital	22	3506	159.36	19	

**Rank**	**Publication titles**	**Number of publications**	**Number of citations**	**Citations per article**	**Impact factor**	

1	Chest	59	4821	81.71	11.393	
2	Internal Medicine	49	421	8.59	1.82	
3	Journal of Heart and Lung Transplantation	36	1446	40.17	13.569	
4	European Respiratory Journal	32	2851	89.09	33.801	
5	Medicine	32	952	29.75	1.817	
6	Seminars in Respiratory and Critical Care Medicine	31	911	29.39	3.921	
7	Chinese Medical Journal	28	249	8.89	6.133	
8	Thorax	28	1608	57.43	9.203	
9	Journal of Cystic Fibrosis	25	799	31.96	5.527	
10	American Journal of Respiratory and Critical Care Medicine	23	2820	122.61	30.528	

**Rank**	**Authors**	**Number of publications**	**Number of citations**	**Citations per article**	**H-index**	

1	Denning D. W.	32	58,544	84.85	104	
2	Agarwal R.	18	9452	21.38	51	
3	Aggarwal A. N.	12	7154	21.04	45	
4	Chakrabarti A.	12	13,084	31.83	58	
5	Husain S.	10	10,214	32	56	
6	Chotirmall S. H.	9	3917	19.68	36	
7	Greenberger P. A.	9	12,405	30.11	52	
8	Kurup V. P.	9	9032	28.49	48	
9	Moss R. B.	9	12,788	43.95	59	
10	Dhooria S.	8	2583	11.9	28	

**Table 2:** The top 10 co-cited references of the Aspergillus in the respiratory system.

Rank	Title	Journal	Country	Author	Year	Number of citations
1	Revised definitions of invasive fungal disease from the European organization for Research and Treatment of Cancer/Invasive Fungal Infections Cooperative Group and the National Institute of Allergy and Infectious Diseases Mycoses Study Group (EORTC/MSG) Consensus Group	Clinical Infectious Diseases	Netherlands	De Pauw B.	2008	125
2	Voriconazole versus amphotericin B for primary therapy of invasive aspergillosis	The New England Journal of Medicine	France	Herbrecht R.	2002	99
3	Treatment of aspergillosis: clinical practice guidelines of the Infectious Diseases Society of America	Clinical Infectious Diseases	USA	Walsh T. J.	2008	84
4	Practice Guidelines for the Diagnosis and Management of Aspergillosis: 2016 Update by the Infectious Diseases Society of America	Clinical Infectious Diseases	USA	Patterson T. F.	2016	73
5	Allergic bronchopulmonary aspergillosis: Review of literature and proposal of new diagnostic and classification criteria	Clinical & Experimental Allergy	India	Agarwal R.	2013	68
6	Clinical and immunological criteria for diagnosis of allergic bronchopulmonary aspergillosis	Annals of Internal Medicine	USA	Rosenberg M.	1977	64
7	Allergic bronchopulmonary aspergillosis in cystic fibrosis-state of the art: Cystic fibrosis foundation consensus conference	Clinical Infectious Diseases	USA	Stevens D. A.	2003	64
8	Allergic bronchopulmonary aspergillosis	Chest	India	Agarwal R.	2009	57
9	A randomized trial of itraconazole in allergic bronchopulmonary aspergillosis	The New England Journal of Medicine	USA	Stevens D. A.	2000	47
10	Invasive Aspergillosis in critically ill patients without malignancy	American Journal of Respiratory and Critical Care Medicine	Belgium	Meersseman, W.	2004	47

## Data Availability

All data generated or analyzed during this study are included in this published article and the original data could be obtained from Web of Science (https://www.webofscience.com/wos/). If any other researcher needs to reproduce data with the help of the raw data we used in our analysis, contact co-corresponding author Fan Jiang (fmmujf@chd.edu.cn).

## Nomenclature

CAPA: COVID-19-associated pulmonary aspergillosis
AIDS: Acquired immune deficiency syndrome
HIV: Human immunodeficiency virus
ISI: Institute for Scientific Information
WoSCC: Web of Science
APHP: Assistance Publique Hopitaux Paris
NEJM: New England Journal of Medicine
LLR: Log-likelihood ratio
FDA: The United States Food and Drug Administration
VHA: Veterans Health Administration
GVHD: Graft-versus-host disease
CML: Chronic myelogenous leukemia
ABPA: Allergic bronchopulmonary aspergillosis
COVID-19: Coronavirus disease
IPA: Invasive pulmonary aspergillosis
EORTC: European Organisation for Research and Treatment of Cancer
MSGERC: Mycoses Study Group Education and Research Consortium
COPD: Chronic obstructive pulmonary disease
GM-CSF: Granulocyte–macrophage colony-stimulating factor
G-CSF: Granulocyte colony-stimulating factor
M-CSF: Macrophage colony-stimulating factor
TNF: Tumor necrosis factor
LFD: Aspergillus-specific lateral flow device
mNGS: Metagenomic next-generation sequencing
BAL: Bronchoalveolar lavage fluid
AFST: Antifungal susceptibility testing
VOCs: Volatile organic compounds
CRISPR: Clustered regularly interspaced short palindromic repeat
cfDNA: Cell-free DNA
